# Management pattern and medication-related harms and its predictors in colorectal cancer patients: an institutional-based retrospective study

**DOI:** 10.3389/fonc.2023.1253845

**Published:** 2023-10-31

**Authors:** Belayneh Kefale, Melaku Tadege Engidaw, Desalegn Tesfa, Mulugeta Molla, Yitayih Kefale, Chernet Tafere

**Affiliations:** ^1^ Clinical Pharmacy Unit and Research team, Department of Pharmacy, College of Medicine and Health Sciences, Bahir Dar University, Bahir Dar, Ethiopia; ^2^ Department of Social and Public Health, College of Health Sciences, Debre Tabor University, Debre Tabor, Ethiopia; ^3^ Pharmacology and Toxicology Unit, Department of Pharmacy, College of Health Sciences, Debre Tabor University, Debre Tabor, Ethiopia; ^4^ Department of Pharmacy, Bahir Dar Health Science College, Bahir Dar, Ethiopia; ^5^ Pharmaceutics Unit and Research team, Department of Pharmacy, College of Medicine and Health Sciences, Bahir Dar University, Bahir Dar, Ethiopia

**Keywords:** colorectal cancer, medication related harm, oncology center, predictors, ethiopia

## Abstract

**Introduction:**

Data on colorectal cancer (CRC) patients’ thorough management practices and medication-related harms (MRH) are scarce. This study’s aim was to investigate the MRHs in patients receiving CRC chemotherapy at the comprehensive specialized hospital of the University of Gondar (UoGCSH).

**Methods:**

A registry-based retrospective cohort study was conducted on CRC patients at the UoGCSH during 2017–2021. From February to May 2022, medical records were reviewed using a pretested data collection tool to collect socio-demographic and disease-related characteristics, MRHs, and medication regimens. MRHs occurrence and adverse drug reactions (ADRs) severity were assessed using standard guidelines and protocols. Version 16/MP of STATA for Windows was used for the analysis. Independent predictors of MRHs were investigated using logistic regression analysis. A p-value ≤0.05 was used to determine an independent variable’s statistical significance.

**Results:**

One hundred forty three CRC patients were included, with a mean age of 49.9 ± 14.5 years. About 32.9% and 33.6% had stage II and III cancer, respectively. Significant patients had co-morbidities (15.4%) and complications (13.3%). Fluorouracil (5-FU)-based regimens were given to more than half (56%) of the patients. MRHs were found in 53.1% of the patients, with a mean of 2.45 ± 1.37 MRHs. The most common MRHs were the need for additional drug therapy, sub-therapeutic dose, DDIs, and ADRs. Being on stage IV (AOR = 27.7, 95% CI = 3.85–199.38, p = 0.001), having co-morbidity (AOR = 7.42, 95% CI = 1.80–30.59, p = 0.018) and having complication (AOR = 11.04, 95% CI = 1.72–70.95, p = 0.011) and treated with five or more drugs (AOR = 2.54, 95% CI = 1.07–6.07, p = 0.035) were independent predictors of MRHs.

**Conclusion:**

A fluorouracil-based treatment regimen was most frequently used. MRHs were found in nearly half of CRC patients. Furthermore, MRHs were significantly associated with cancer stage, comorbidity and complication status, and the number of medications used. Because MRHs are common, improving clinical pharmacy services is critical for optimizing drug therapy in CRC patients.

## Introduction

Although developed countries have a higher cancer burden, mortality rates in developing countries are much higher (1). A cancer of the large intestine is colorectal cancer (CRC) (2, 3).

CRC is a significant global health concern, with a high incidence and mortality rate. According to recent data from the World Health Organization (WHO) (4), CRC ranks as the third most common cancer worldwide, affecting millions of individuals annually. Furthermore, a study done by Douaiher et al. (1), highlights that CRC is the second leading cause of cancer-related deaths.

The incidence of CRC was found to be 4.04 per 100,000 people in Sub-Saharan Africa, with a male-to-female ratio of 1.2:1 and an estimated 24,711 new cases reported annually (5). In Ethiopia, it affects men the most frequently (6). Unfortunately, a 6-year retrospective cohort study of CRC patients in Ethiopia revealed a mortality rate of 34.8% (7).

Treatment for CRC patients is complex and carries an inherent risk of MRHs, which ultimately influences treatment outcome due to the high prevalence of co-occurring chronic diseases (8). The stage of CRC at diagnosis and the location of the tumor influence treatment. The most typical therapy for early-stage (stage I or II) CRC is surgical removal of the tumor and any adjacent lymph nodes. Chemotherapy alone or in conjunction with radiation therapy is frequently administered prior to or following surgery for individuals with late-stage illnesses (9). The existence and kind of comorbidities, drug therapy problems, screening practices, and treatment accessibility all contribute to worse treatment results (10, 11).

A MRH is described as “an event or scenario involving medication therapy that actually or potentially interferes with anticipated health outcomes” by the Pharmaceutical Care Network of Europe (PCNE) (12). The likelihood of developing MRHs like ADRs, drug interactions, medication errors, and non-compliance increases with drug therapy complexity (13). Up to 25% of hospitalized patients have been documented to have ADRs, which may be made worse by unneeded pharmaceutical therapy, improper drug selection, and untreated conditions. Significant morbidity and mortality can result from MRHs (14). MRHs in cancer chemotherapy can have negative effects due to the anticancer medicines’ high toxicity and narrow therapeutic window (13).

Cancer patients are particularly vulnerable to drug interactions due to their exposure to anticancer drugs and frequently experience comorbid illnesses and tumor-related symptoms like pain, depression, and seizures. Drug pharmacokinetics in cancer patients are altered for a variety of hypothetical reasons, including drug interactions with liver enzymes, impaired drug excretion in patients with renal and/or hepatic dysfunction, hampered drug absorption due to mucositis, malnutrition, and infection, and variation in the volume of drug distribution due to decreased levels of serum binding proteins (15). A more thorough investigation of MRHs in CRC patients might offer invaluable information to healthcare professionals regarding MRH management and/or prevention (16). Several studies have found that DRPs cause significant hospitalizations, with 50% of them being avoidable, and have a significant negative impact on the health of cancer patients (17–19). Studies in Ethiopia (20), India (21) reported the prevalence of MRHs caused by chemotherapy in cancer patients was 48.7%and 58.6%, respectively. Various studies showed that adverse drug reaction (ADR), the need for additional drug therapy, and drug–drug interactions (DDI) are the most prevalent DRPs (20, 22, 23). Several studies have found that MRH development is influenced by sex, age, length of hospital stay, cancer stage, polypharmacy, co-morbidity, and complication status (20, 24, 25). Data on thorough MRHs among CRC patients are, however, rare. The majorities of studies that have been published thus far have either addressed the issue of drug-related hospital admissions or have exclusively looked at ADRs among hospitalized patients. A comprehensive MRH study would provide valuable insight for healthcare providers in reducing the incidence of MRHs and improving treatment outcomes in cancer patients. A systematic review found that MRHs can be prevented and managed with the help of clinical pharmacists (26, 27). Thus, this study aimed to examine the MRHs in patients receiving CRC chemotherapy and management pattern at the UoGCSH.

## Methods

### Study design and setting

This study was a registry-based retrospective cohort using the UoGCSH data collected from February to May 2022. UoGCSH is located in the outskirts of the city of Gondar serving more than forty million inhabitants of Amhara Regional State as an oncologic center. The oncology center comprises a range of professionals providing treatment for different cancer types, including CRC and its complications.

### Study participant

All histologically confirmed adult CRC patients’ charts/registries, or logbooks who were receiving chemotherapy at the UoGCSH oncology center from 2017 to 2021 were part of this study.

### Operational definition

Medication-related harm: In our study, MRH describes at least one of the following undesirable events: unsafe, ineffective, DDI, medication use without indication, and the need for additional drug therapy (17).

### Sampling techniques and data collection tool

A total survey sampling technique was employed to select 143 medical records with confirmed colorectal cancer. The data collection instrument was created using peer-reviewed published journal findings and the MRH lists of Cipole et als (12, 23, 28). The format contains socio-demographic such as sex, age and residence and disease-related characteristics, including but not limited to: histological types and stages of cancer, presence and types of complications and comorbidities, treatment modalities, and status and list MRHs. The patient’s age was collected as a continuous variable and categorized for analysis purposes.

The European Society for Medical Oncology practice guideline, the NCCN, and the Ethiopian cancer treatment protocols were employed to evaluate the occurrence of MRHs. Stockley’s Drug Interactions Checker and the modified Hartwig and Siegel ADR Severity Assessment Scale were used to assess the occurrence of DDIs and the severity of ADRs, respectively (29).

### Recruitment of data collectors

Six data collectors were hired: three nurses and three pharmacists. The data collectors received pretest training one day before the commencement of data collection, focusing on the data collection tool, research ethics, selection criteria, and confidentiality.

### Data quality control

Before starting data collection, a pretest was performed on the medical records of eight CRC patients to ensure that the data abstraction format was understandable. The results of the pretest were used to make changes. Furthermore, the data collection process was closely monitored on-site throughout the data collection period, and the data’s completeness and consistency were checked on a daily basis.

### Data analysis

After a week of data collection, data analysis was carried out. EpiData 4.6 for Windows was used to prep, verify, code, and enter the data before exporting it to STATA version 16/MP for analysis. A bivariable logistic regression analysis was performed to investigate whether there is an association between the occurrence of MRH and various independent factors. All variables such as sex, age, co-morbidity and complication status, cancer stage, and number of medications with p<0.2 in the bivariable logistic regression analysis were included in the multivariable logistic regression. A 95% CI was constructed for Adjusted Odds Ratios (AORs) to determine the strength of associations. The statistical significance of each independent variable was determined using a p-value ≤ 0.05.

## Results

### Socio-demographic and clinical characteristics of study participants

The study included 143 CRC patients with a mean age of 49.9 ± 14.5 years. Males (61.5%) and urban residents (58.7%) made up nearly two-thirds of study participants. Adenocarcinoma (86.7%) was the most common histological type in terms of clinical characteristics. This study also showed that two-thirds of the participants had stage II or III cancer. Furthermore, one-fourth of the patients had metastases, with the liver and lung being the most common metastatic sites. Less than one-fifth of patients had co-morbidities and complications, with hypertension and anemia being the most common co-morbid conditions and complications, respectively (**Table 1**).

**Table 1:** Study participants of Socio-demographic and clinical characteristics.

Variables	Category	Frequency	Percent
Sex	Female	55	38.5
	Male	88	61.5
Age	25-40	46	32.2
	41-50	35	24.5
	≥51	62	43.4
Residence	Rural	59	41.3
	Urban	84	58.7
Histological cell type	Squamous Cell Carcinoma	13	9.1
	Adenocarcinoma	124	86.7
	Not documented	6	4.2
Stages of cancer	Stage I	12	8.4
	Stage II	47	32.9
	Stage III	48	33.6
	Stage IV	36	25.2
Recurrence status	Yes	12	8.4
	No	131	91.6
Metastasis status	Yes	36	25.2
	No	107	74.8
Sites of metastasis	Liver	15	41.7
	Lung	5	13.9
	Ovary + liver	4	14.3
	Lymph node	4	11.1
	Lung + liver	3	8.3
	Lung + liver + Lymph node + peritoneum	3	8.3
	Lymph node + bone	2	5.6
Co-morbidity status	Yes	22	15.4
	No	121	84.6
List of co-morbidities	Hypertension	8	36.4
	Tonsillopharyngitis	4	18.2
	Stomach prolapses	3	13.6
	Sepsis	3	13.6
	Chronic kidney disease	2	9.1
	Benign prostatic hyperplasia	1	4.5
Complication status	Yes	19	13.3
	No	124	86.7
List of complications	Anemia	10	52.6
	Intussusception	3	15.8
	Hydronephrosis	3	15.8
	Septic shock	3	15.8

### Medication pattern and types of chemotherapeutic regimens

About two-thirds (67.1%) of the patients were on chemotherapy alone. Metoclopramide with dexamethasone (52, 36.4%) combination were the most commonly used prophylactic antiemetic regimen followed by a combination of ondansetron and dexamethasone (34, 23.8%). However, one-fourth of patients did not receive antiemetic prophylaxis. According to the study’s findings, the most commonly used analgesics were morphine (28%), paracetamol (24%), and diclofenac (20.3%) (**Table 2**).

**Table 2:** Medications used for colorectal cancer patients.

Variables	Category	Frequency	Percent
Number of medications	<5	60	42
	≥5	83	58
Prophylactic antiemetic regimens	Metoclopramide and dexamethasone	52	36.4
	Ondansetron and dexamethasone	34	23.8
	Metoclopramide	11	7.7
	Ondansetron	9	6.3
	No-antiemetic’s given	37	25.9
Analgesics regimens	Morphine	40	28.0
	Paracetamol	34	24.0
	Diclofenac	29	20.3
	Tramadol	22	15.4
	Ibuprofen	6	4.2
	Analgesic not given	23	16.1
Treatment modalities	Chemotherapy	96	67.1
	Chemotherapy + surgery	34	23.8
	Surgery	10	7.0
	Chemotherapy + radiotherapy	3	2.1

**Figure 1:**
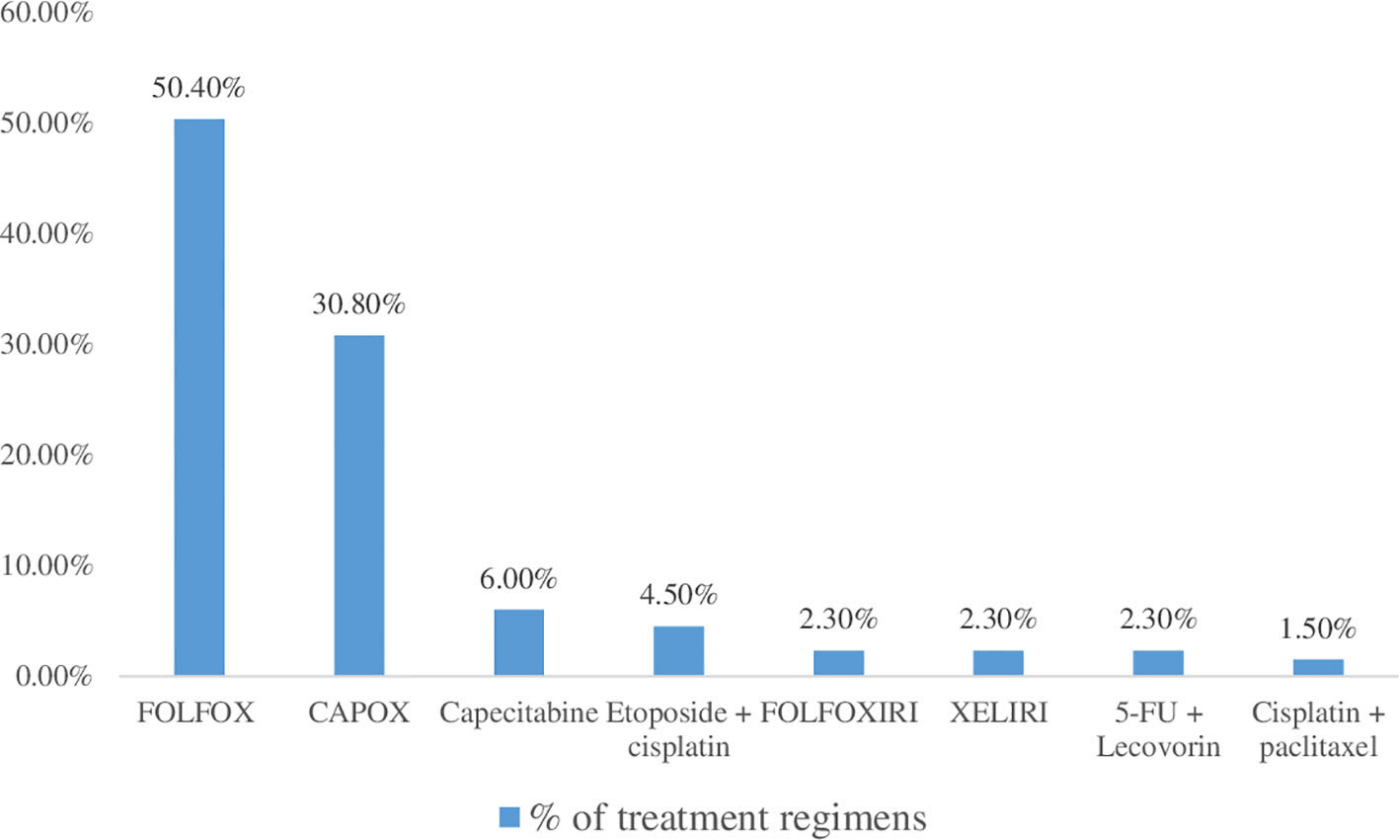
Chemotherapy’s used in the treatment of colorectal cancer at UoGCSH.

The combination of cisplatin, *5-FU* and oxaliplatin (50.4%) followed by oxaliplatin and capecitabine (30.8%) were the most widely used treatment regimen. The combination of cisplatin and paclitaxel, however, was the least commonly used treatment regimen (**Figure 1**).

### Medication-related harms and associated factors

A total of 186 MRHs were found in 76 colorectal cancer patients, representing 53.1% prevalence. A mean of 2.45 ± 1.37 MRHs per patient occurred. The most common MRHs were the need for additional drug therapy, sub-therapeutic doses, and DDIs, accounting for 29.6%, 22%, and 18.3% of cases, respectively (**Table 3**).

**Table 3:** Types of medication-related harms among patients with colorectal cancers.

Types of MRHs	Frequency (n)	Percent (%)
Need for additional drug therapy	55	29.6
Sub-therapeutic dose	41	22.0
Drug interaction	34	18.3
Adverse drug reaction	34	18.3
Medication use without indication	13	7.0
Overdose	9	4.8

**Figure 2:**
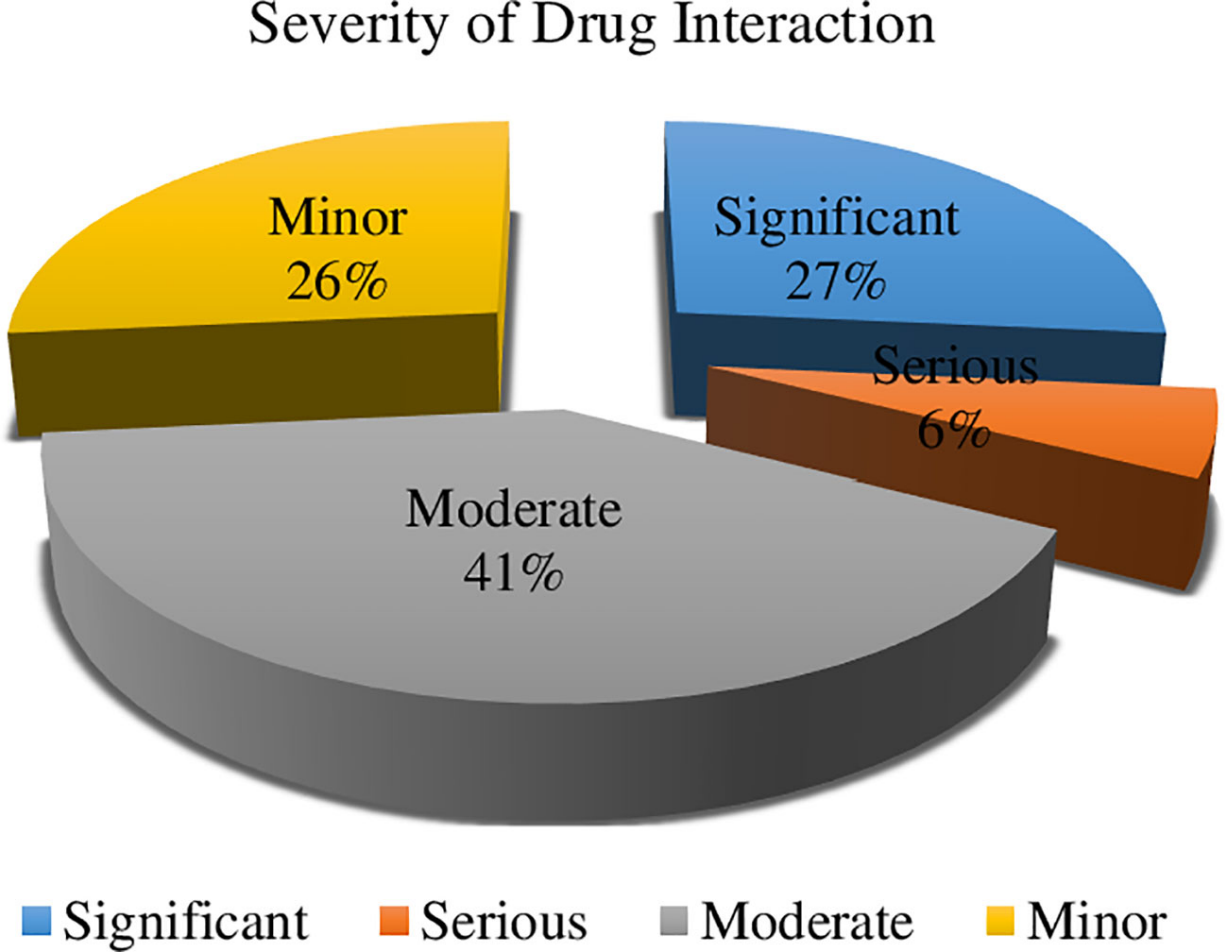
Severity of drug interactions among colorectal cancer patients at UoGCSH (n = 34).

In terms of the severity of drug interactions, 41% were moderate, necessitating close monitoring of the drug interactions’ outcomes. Ciprofloxacin interacting with oxaliplatin, metoprolol combined with hydrochlorothiazide, and metoprolol in conjunction with ibuprofen were some of the noteworthy drug-drug interactions that were noticed. However, 6% of them were severe and required the use of alternative medications (**Figure 2**). In our study, we identified serious drug interactions, for example, between ciprofloxacin and ondansetron.

Nausea (61.8%), leucopenia (50%), and vomiting (47.1%) were the most frequent types of ADRs. Conversely, thrombocytopenia (2.9%) was the least frequent ADR (**Table 4**).

**Table 4:** Adverse drug reactions in the study participants (n = 34).

Adverse drug reaction	N (%)
Nausea	21 (61.8)
Leucopenia	17 (50)
Vomiting	16 (47.1)
Dizziness	13 (38.2)
Neutropenia	9 (26.5)
Constipation	3 (8.8)
Thrombocytopenia	1 (2.9)

### Predictors of medication related harms

The stage of cancer, co-morbidity and complication status, and the number of medications all had a significant association with the development of MRHs in our study, as identified by multivariable logistic regression analysis. CRC patients in stage IV were 28 times (AOR = 27.7, 95% CI = 3.85–199.38, p = 0.001) more likely to have MRHs than CRC patients in stage I. In addition, when compared to their counterparts, CRC patients with co-morbidity and complications were 7 times (AOR = 7.42, 95% CI = 1.80–30.59, p = 0.018) and 11 times (AOR = 11.04, 95% CI = 1.72-70.95, p = 0.011) more likely to develop MRHs. Patients who had taken five or more medications were three times more likely to develop MRHs (AOR = 2.54, 95% CI = 1.07–6.07, p = 0.035) than those who had taken fewer than five medications (**Table 5**).

**Table 5:** Univariable and multivariable binary logistic regression analysis of medication-related harm predictors.

Variables		COR (95% CI)	p-value	AOR (95% CI)	p-value
Sex	Female	1		1	
	Male	2.11 (1.06–4.18)	0.033	1.77 (0.72–4.36)	0.214
Age	25-40	1		1	
	41-50	0.18 (0.07–0.49)	0.001	0.25 (0.07–1.17)	0.052
	>51	0.87(0.39–1.94)	0.739	0.83 (0.27–2.60)	0.748
Co-morbidity	No	1		1	
	Yes	4.89 (1.56–15.29)	0.006	7.42 (1.80–30.59)	0.006
Complications	Yes	9.36 (2.08–42.26)	0.004	11.04 (1.72–70.95)	0.011
	No	1		1	
Stages of cancer	I	1		1	
	II	1.36 (0.36–5.15)	0.654	4.01 (0.74–21.79)	0.108
	III	1.56 (0.41–5.88)	0.515	2.81 (0.53–14.90)	0.226
	IV	16.00 (3.27–78.28)	0.001	27.70 (3.85–199.38)	0.001
Number of medications	<5	1		1	
	≥5	4.15 (2.05–8.41)	<0.001	2.54 (1.07–6.07)	0.035

## Discussion

Patients with CRC are at high risk for MRH due to the complexity of the management pattern and the presence of various socio-demographic and clinical factors associated with the development of MRH. In the present study the male-to-female ratio for CRC patients in this study was 1.6:1, which is consistent with studies conducted in Tanzania, the United Kingdom, and China (2, 30–32) but differs from previous studies with a very small male preponderance for CRC (33, 34). Many biological and behavioral factors may contribute to men’s increased susceptibility to CRC (35–38). Moreover, men are more likely to store visceral fat (39), which has been linked to a higher risk of CRC (40–42).

The most prevalent histological type (86.7%) was adenocarcinoma, which was consistent with several findings (30, 33, 43–47). In the current study, there were 58.8% of patients with advanced CRC (stage III, 33.6%, and stage IV, 25.2%), which was greater than what was shown in other studies (33, 46). A typical observation in underdeveloped nations is that the majority of patients are detected at an advanced stage (48, 49). This might be as a result of the fact that most cancer patients in developing nations like Ethiopia seek treatment extremely late in the course of their illness. Early-onset CRC has been found to be on the rise recently in a number of nations (50–52).

Anemia (52.6%) was the most frequent co-occurring complication, affecting about 13.3% of patients. Similar to this, anemia is a condition that affects a significant number of colorectal patients (20, 53, 54) and is associated with a worse prognosis (55).

Regarding management pattern, a three-drug combination (5-FU plus leucovorin plus Oxaliplatin, or FOLFOX), followed by a two-drug combination (Oxaliplatin plus Capecitabine, or XELOX), which is frequently used in Ethiopian cancer centers, was taken by the majority (50.4%) of CRC patients who are receiving only chemotherapy. This is also recommended by the American Cancer Society and the National Comprehensive Cancer Network, and it is consistent with an Ethiopian and Kenyan research (20, 33, 48, 49). Combination chemotherapeutic treatments have been demonstrated to increase quality of life, time to progression, and overall survival while free of illness (9). This might be attributable to the synergistic and additive effects of chemotherapy medication combinations. These medications work in a variety of ways to help kill malignant cells (56).

MRHs are major healthcare problems, and a large proportion of them can be avoided. A total of 186 MRHs were found in 76 CRC patients, resulting in 53.1% prevalence. This finding is in line with another Ethiopian study (20) that demonstrates 48%. Contrastingly, when compared to prospective research conducted in Kenya that revealed 132 MRHs in 71 CRC patients, which was much greater (33). These differences suggest that comparing the results is challenging due to variations in study contexts, measurement methods, and classification systems. The risk of developing MRHs like ADRs, comorbidities, drug interactions, and non-adherence increases with the complexity of the chemotherapy (14). The most frequent MRHs were the need for additional drug therapy (29.6%), sub-therapeutic dose (22%), drug interaction (18.3%), and ADRs (18.3%). On the other side, a study done in Kenya found that ADRs, the need for additional drug therapy, and non-compliance were the most frequent MRHs (33).

Adverse drug events are frequent in chemotherapy patients due to the drug’s pharmacodynamic properties and narrow therapeutic indices (17). Many ADRs seem inevitable because most cytotoxic drugs cannot distinguish between healthy and cancerous cells. Nausea and leucopenia were the most frequently reported adverse drug reactions, at 61.8% and 50%, respectively. Serotonin plays an important role in both acute and delayed chemotherapy-induced nausea and vomiting, involving both peripheral and central nervous system pathways (57).

In our study, taking five or more medications was found to be an independent predictor of the presence of MRHs. Gender and age were not predictors of the presence of MRHs in a study conducted in Singapore and at Tikur Anbessa Specialized Hospital, which is consistent with our findings (17, 58). Puts et al. discovered that being ≥76 years old and taking ≥5 drugs were risk factors for moderate-to-severe potential MRH when they investigated the association between patient factors and the existence of MRH in an older cancer cohort (58). The fact that this study focuses on a specific patient population and involves clinical pharmacists and oncology nurses in data collection, which is critical to ensuring data quality, is one of its strengths. Numerous studies have demonstrated clinical pharmacists’ ability to recognize and prevent clinically important MRHs, as well as physicians’ recognition and responsiveness to clinical pharmacist recommendations for drug-related interventions (59, 60). Our study has two significant limitations: reliance on patient medical charts for retrospective analysis and a single-site design focused on patients at the UoGCSH oncology center. Consequently, the results may not be generalizable to the broader population and should be interpreted cautiously. Thus, multicenter prospective interventional follow-up studies are required.

## Conclusion

FOLFOX and XELOX treatment regimen were most frequently used. MRHs were identified in 53.1% of CRC patients. The study revealed that 29.6% of patients experienced the need for additional drug therapy, while 22% received sub-therapeutic doses. Furthermore, the stage of cancer, the presence of comorbidity and complications, and the number of medications taken were all independent predictors of developing MRHs. Given the prevalence of MRHs, it is crucial to enhance clinical pharmacy services and medication review stewardship to maximize the effectiveness of CRC treatment.

## Data availability statement

The original contributions presented in the study are included in the article/supplementary material, further inquiries can be directed to the corresponding author/s.

## Ethics statement

The studies involving humans were approved by Debre Tabor University Ethical Review Board. The studies were conducted in accordance with the local legislation and institutional requirements. Written informed consent for participation was not required from the participants or the participants’ legal guardians/next of kin in accordance with the national legislation and institutional requirements.

## Author contributions

BK: Conceptualization, Data curation, Formal Analysis, Funding acquisition, Investigation, Methodology, Project administration, Resources, Software, Supervision, Validation, Visualization, Writing – original draft, Writing – review & editing. ME: Conceptualization, Formal Analysis, Funding acquisition, Methodology, Writing – original draft. DT: Conceptualization, Data curation, Formal Analysis, Writing – review & editing. MZ: Methodology, Software, Validation, Visualization, Writing – review & editing. YK: Data curation, Formal Analysis, Investigation, Methodology, Visualization, Writing – original draft. CT: Methodology, Validation, Visualization, Writing – original draft, Writing – review & editing.
